# Development and validation of a mobile health application usability scale for older adults with chronic diseases

**DOI:** 10.1093/geroni/igaf122.3159

**Published:** 2025-12-31

**Authors:** Hongyu Yu, Weiyu Qiu, Yanfeng Wang, Qinyang Wu, Ke Hu, Qiaohong Yang

**Affiliations:** University of Washington, Seattle, Washington, United States; The First Affiliated Hospital of Jinan University, Guangzhou, Guangdong, China (People’s Republic); Jinan University, Guangzhou, Guangdong, China (People’s Republic); Jinan University, Guangzhou, Guangdong, China (People’s Republic); Jinan University, Guangzhou, Guangdong, China (People’s Republic); Jinan University, Guangzhou, Guangdong, China (People’s Republic)

## Abstract

**Background:**

Chronic diseases are a leading cause of disability and death in older adults. Mobile health (mHealth) applications can improve chronic disease management and reduce healthcare costs, but many are not designed for older users. A reliable tool is needed to assess mHealth usability for this population.

**Objective:**

To develop and validate an mHealth usability evaluation scale for older adults with chronic diseases.

**Methods:**

The scale was developed (March–September 2022) through literature review, interviews, team discussions, and the Delphi method. A pilot test refined the scale, followed by surveys in Guangzhou, China (October–December 2022), using Item Discrimination Index, correlation analysis, internal consistency tests, and exploratory factor analysis. Reliability and validity evaluations were conducted from October 2022 to February 2023.

**Results:**

The final scale included six dimensions and 23 items. Content validity indices ranged from 0.85–1. Fit indices were strong (χ²/df = 1.728, fit indices = 0.817–0.928). Average Variance Extraction (>0.4) and Composite Reliability (>0.6) met criteria. Cronbach’s α ranged from 0.758–0.911, indicating good internal consistency.

**Conclusions:**

The usability scale was rigorously developed and demonstrated strong reliability and validity. It provides a valuable tool for assessing mHealth usability among older adults, supporting age-friendly digital health solutions.

